# Serum Lactate Could Predict Mortality in Patients With Spontaneous Subarachnoid Hemorrhage in the Emergency Department

**DOI:** 10.3389/fneur.2020.00975

**Published:** 2020-09-04

**Authors:** Chang Hwan Oh, Jong Won Kim, Geon Ha Kim, Kyeong Ryong Lee, Dae Young Hong, Sang O Park, Kwang Je Baek, Sin Young Kim

**Affiliations:** ^1^Department of Emergency Medicine, Konkuk University Medical Center, School of Medicine, Konkuk University, Seoul, South Korea; ^2^Research Institute of Medical Science, Konkuk University School of Medicine, Seoul, South Korea; ^3^Department of Neurology, College of Medicine, Ewha Womans University Mokdong Hospital, Ewha Womans University, Seoul, South Korea

**Keywords:** lactate, subarachnoid hemorrhage, aneurysm, mortality, emergency department

## Abstract

**Background:** Serum lactate is a useful biomarker for prediction of mortality in critically ill patients. The purpose of this study was to identify if serum lactate could be used as a biomarker for predicting mortality in patients with subarachnoid hemorrhage (SAH) in the emergency department.

**Methods:** This retrospective study enrolled 189 patients. Baseline demographic data and clinical characteristics of patients were obtained from medical record review. Multiple logistic regression analysis was performed to determine predictor variables significantly associated with mortality. Receiver operating characteristic (ROC) curve analysis was used to evaluate the performance of variables for mortality prediction in SAH.

**Results:** Using multivariate logistic regression analysis, age [OR 1.05; 95% confidence interval (CI) 1.00–1.10; *p* = 0.037], Hunt and Hess scale score (OR 3.29; 95% CI 1.62–6.70; *p* = 0.001), serum lactate level (OR 1.33; 95% CI 1.03–1.74; *p* = 0.032), and serum glucose level (OR 1.01; 95% CI 1.00–1.02; *p* = 0.049) predicted overall mortality in SAH. The area under the ROC curve (AUC) value for the use of serum lactate level to predict mortality in SAH was 0.815 (95% CI 0.753–0.868) (*p* < 0.001).

**Conclusion:** Serum lactate may be a useful biomarker for the early prediction of mortality in SAH patients in the emergency department.

## Introduction

A spontaneous subarachnoid hemorrhage (SAH) occurring secondary to a ruptured cerebral aneurysm is a devastating neurological disorder accounting for 5% of all stroke subtypes (1). About one-third of patients with this disorder die within a few days of initial bleeding, and most survivors have long-term neurological disability (2). Although surgical and medical advances have improved overall outcomes in these patients, SAH still remains a serious disease with a high mortality rate. Prediction of patient outcomes also remains challenging.

In the emergency department (ED), early and reliable prediction of outcomes for SAH can guide clinicians during treatment decision-making and can provide patients and their families with sufficient information about the disease. Prognostic determinations in patients with SAH are generally based on clinical and radiologic evaluations. The Hunt and Hess grading scale and the World Federation of Neurosurgical Societies grading scale are commonly used clinical tools for prediction of outcomes in these patients (3, 4). The modified Fisher grading scale is a radiology-based tool for predicting risks of clinical vasospasm and delayed cerebral ischemia, as well as patient outcomes (5). Recently, several biomarkers have been proposed as potential early predictors of disease prognosis in the ED. A reliable biomarker, in conjunction with clinical and radiologic scales, may be helpful for prognostic predictions in patients with SAH.

Serum lactate is a readily available biomarker in most EDs and has been shown to provide useful information about disease severity and mortality risk in critically ill patients (6). Moreover, its role in prognostic predictions for patients with sepsis and trauma has also been well-established (7, 8). However, comparatively little research has been devoted to the use of serum lactate in the management of patients with SAH. In this study, we hypothesized that serum lactate would be associated with outcomes in SAH patients, and we aimed to identify whether serum lactate could be used as a biomarker for predicting mortality in SAH patients in the ED.

## Methods

### Study Design and Subjects

This retrospective study was conducted through analysis of medical records from consecutive patients with SAH who presented to the ED of an urban hospital in Seoul, Korea from March 2013 to October 2017. The Institutional Review Board approved this study, and the need for informed consent was waived. We included patients older than 18 years of age with SAH caused by ruptured aneurysms. We excluded patients with non-aneurysmal SAH, patients presenting with cardiac arrest, and patients who did not have a serum lactate obtained in the ED.

We obtained baseline demographic and clinical data, including patient age, gender, vital signs, medical history, and comorbidities, from the medical record. The clinical grade at presentation, assessed using the Hunt and Hess scale, and the radiographic grade, assessed using the modified Fisher scale, were also collected. Results from routine blood tests performed at presentation, including serum lactate level, were reviewed. All patients were treated according to the standard protocol, and the primary outcome of our study was overall mortality.

### Data Analysis

All results are presented as medians with interquartile range (IQR) for continuous variables and numbers with percentages for categorical variables. Continuous variables were compared using the Mann-Whitney *U*-test, and categorical variables were compared using the chi-squared test. Univariate analysis was performed to identify relationships between predictor variables and outcome. Multiple logistic regression analysis with stepwise backward elimination of independent variables identified to be significant in univariate analysis was performed to determine the predictor variables associated with mortality. Results are presented as odds ratios (OR) with corresponding 95% confidence intervals (CI).

We also performed receiver operating characteristic (ROC) curve analysis to assess the use of serum lactate level for mortality prediction in patients with SAH, and area under the curve (AUC) values were computed. A *p* < 0.05 was considered to be statistically significant. All statistical analyses were performed using SPSS 19 (version 19.0, Seoul, Korea).

## Results

**Figure 1:**
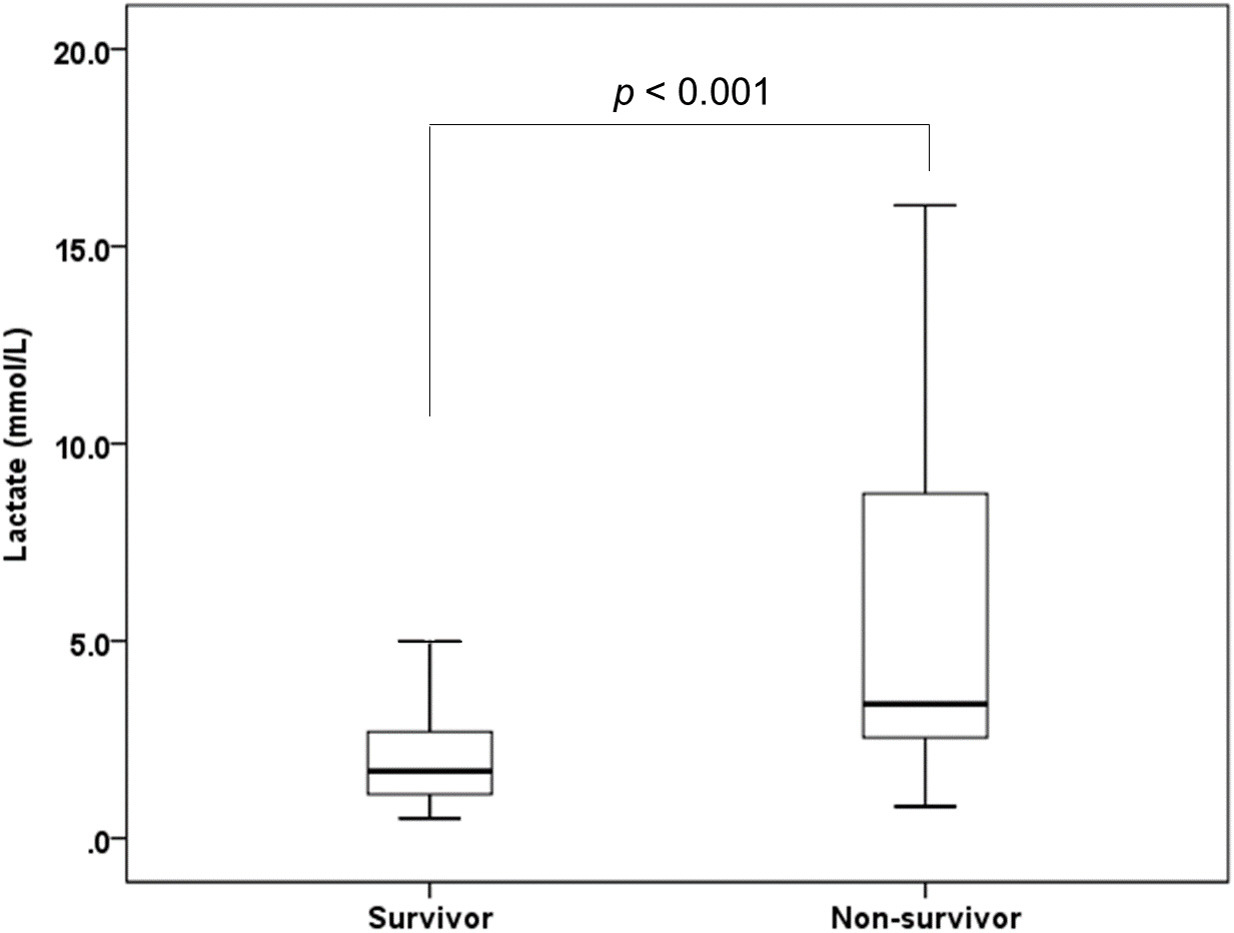
Box and Whisker plot illustrating distribution of serum lactate level between survivor group and non-survivor group of SAH.

A total of 223 patients were considered for inclusion in this study. Of these, 25 patients were excluded because they did not have a reported serum lactate level, and nine patients were excluded because they presented with cardiac arrest. A total of 189 patients were enrolled in the study, with 165 (87.3%) patients survived whereas a total of 24 patients died. The estimated time from SAH to death of the participants was 4 (IQR: 2–7) days. The number of patients whose time to death was over a week was 7 (29.1%). Baseline characteristics of the patients are presented in Table 1. The serum lactate level was 1.7 (IQR: 1.1–2.7) mmol/L in the survival group and 3.4 (IQR: 2.5–8.9) mmol/L in the non-survival group (*p* < 0.001) (Figure 1).

**Table 1:** Baseline Characteristics of the patient.

	**Survivor**	**Non-survivor**	***p*-value**
Age (yr)	53 (46–62)	59.5 (49.8–71.8)	0.038
Gender (M)	76 (46.1%)	8 (33.3%)	0.241
GCS	14 (12–15)	6 (3–7.8)	<0.001
Hunt-Hess grade	2 (2,3)	4 (4,5)	<0.001
Modified Fisher grade	3 (3,4)	4 (4)	<0.001
Aneurysm size (cm)	4.3 (3.4–6)	4.2 (3–6.3)	0.551
Aneurysm location			0.622
Anterior cerebral artery	75 (45.5%)	8 (33.3%)	
Middle cerebral artery	36 (21.8%)	5 (20.8%)	
Posterior cerebral artery	40 (24.2%)	8 (33.3%)	
Other	14 (8.5%)	3 (12.5%)	
Seizure before presentation	11 (6.7%)	3 (12.5%)	0.308
SBP (mmHg)	151 (135–175)	142 (96.8–170)	0.244
HTN	59 (35.8%)	9 (37.5%)	0.868
DM	8 (4.8%)	4 (16.7%)	0.027
WBC (cell/ul)	10,990 (8,460–13,930)	12,875 (10,545–19497.5)	0.004
CRP (mg/dL)	0.07 (0.04–0.19)	0.08 (0.03–0.32)	0.705
Lactate (mmol/L)	1.7 (1.1–2.7)	3.4 (2.5–8.9)	<0.001
Glucose (mg/dL)	143 (124–170)	204 (154.2–296.8)	<0.001
Creatinine (mg/dL)	0.78 (0.69–0.92)	0.91 (0.73–1.26)	0.037

Predictor variables identified in univariate analysis are shown in Supplemental Table 1. In multivariate logistic regression analysis, age (OR 1.05; 95% confidence interval (CI) 1.00–1.10; *p* = 0.037), Hunt and Hess scale score (OR 3.29; 95% CI 1.62–6.70; *p* = 0.001), serum lactate level (OR 1.33; 95% CI 1.03–1.74; *p* = 0.032), and serum glucose level (OR 1.01; 95% CI 1.00–1.02; *p* = 0.049) significantly predicted overall mortality in SAH (Table 2).

**Table 2:** Multivariate analysis of various factors for predicting mortality in SAH.

	**Multivariate analysis**		
	**OR**	**95% CI**	***p-*value**
Age (yr)	1.05	1.00–1.10	0.037
Hunt-Hess scale	3.29	1.62–6.70	0.001
Lactate (mmol/L)	1.33	1.03–1.74	0.032
Glucose (mg/dL)	1.01	1.00–1.02	0.049

**Figure 2:**
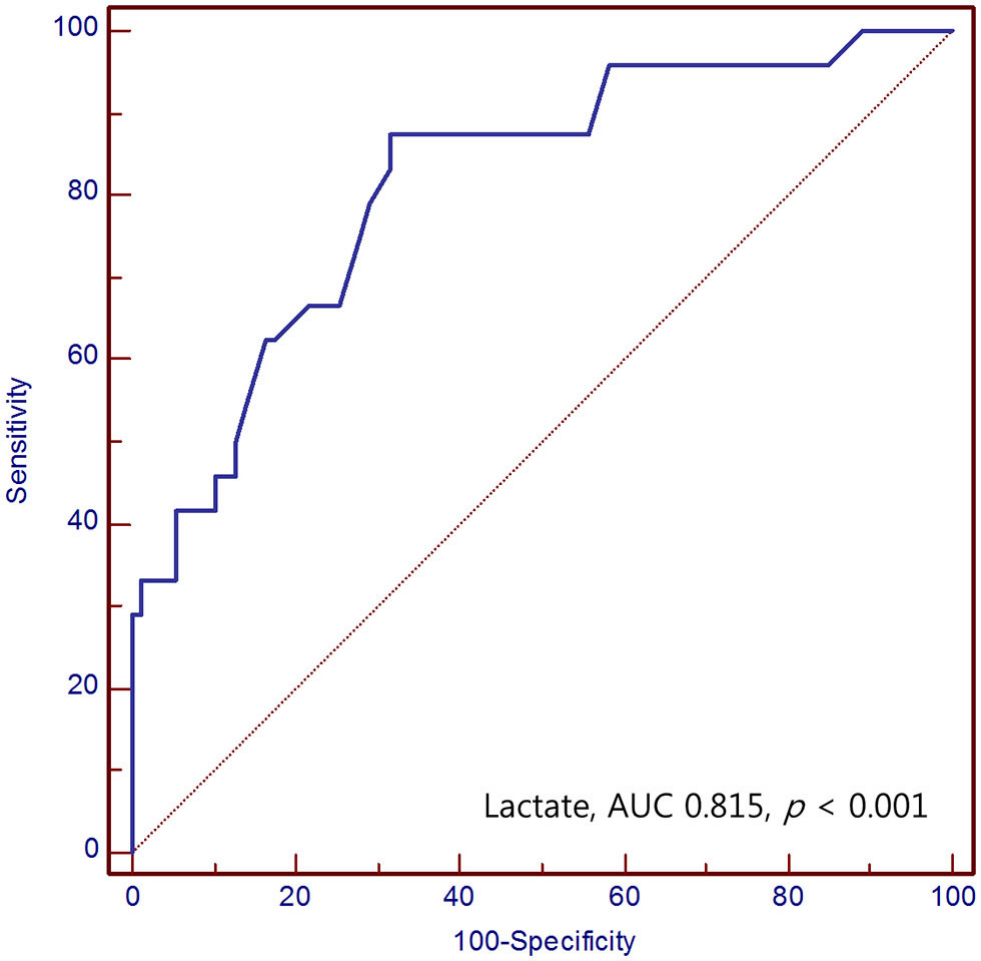
Receiver operating characteristic curve of the serum lactate for predicting mortality of SAH patients in the emergency department.

The area under the ROC curve (AUC) value of serum lactate for prediction of mortality in SAH was 0.815 (95% CI 0.753–0.868) (*p* < 0.001). The optimal cut-off value for serum lactate was 2.3 mmol/L, with a sensitivity and specificity of 87.5 and 68.5%, respectively (Figure 2).

## Discussion

In this study, we evaluated the performance of serum lactate level for prediction of mortality in patients with SAH in the ED. Our study demonstrated that the serum lactate level was significantly higher in the non-survival group than in the survival group. We also demonstrated that the serum lactate obtained in the ED and the clinical grade of SAH were significantly associated with mortality. This finding suggests that serum lactate is a reliable predictor of mortality in patients with SAH and that an elevation of the serum lactate level to only > 2.3 mmol/L might be considered critical in these patients. Although the specificity of the serum lactate was relatively low in our study, the cut-off level of serum lactate above 2.3 demonstrated high sensitivity enough to predict mortality of SAH. This finding suggests that the serum lactate could be a suitable marker for “screening” initial prognosis for the mortality of SAH in the emergency department. However, further studies regarding prognostic markers with high sensitivity and specificity for SAH patients should be warranted.

The mechanism for the elevated lactate level in these patients remains unclear. It is well-known that tissue hypoxia and/or oxygen debt secondary to hypoperfusion are major factors contributing to elevations in blood lactate concentrations (8, 9). If cells experience an insufficient oxygen supply, oxidative phosphorylation in mitochondrial respiration is significantly inhibited and lactate is not oxidized, subsequently leading to its accumulation (10). In addition, hypoxia triggers anaerobic glycolysis, a process in which pyruvate is converted to lactate by lactate dehydrogenase rather than being used in the acetyl-CoA pathway. Therefore, a large amount of lactate is produced under conditions of tissue hypoxia and anaerobic metabolism (11, 12).

Interestingly, most SAH patients in this study did not experience severe hypotension or profound hypoxia in the ED. The elevated serum lactate concentration observed in patients with SAH might also therefore be explained by mechanisms other than tissue hypoxia.

Contrary to the traditional belief that the elevated blood lactate concentration observed in critically ill patients is a byproduct of anaerobic glycolysis, a recent study demonstrated an independent relationship between lactate and tissue hypoxia (13, 14). Lactate can also be produced by accelerated aerobic glycolysis (12). The glycolytic pathway is linked to the activity of the Na^+^/K^+^-ATPase pump. Epinephrine increases cyclic AMP levels through β_2_-adrenergic receptor activation (14). This increase stimulates glycogenolysis and Na^+^/K^+^-ATPase pump activation. Stimulation of the Na^+^/K^+^-ATPase pump generates ADP, which increases phosphofructokinase activity and accelerates aerobic glycolysis, hence producing more pyruvate and, consequently, more lactate (11, 15, 16). Levy et al. demonstrated that exaggerated aerobic glycolysis in skeletal muscle leads to lactate production in septic shock. In addition, infusion of ouabain, a specific inhibitor of Na^+^/K^+^-ATPase, stopped muscle lactate production (17).

Meanwhile, marked sympathetic nervous system activation can occur in patients with SAH (18). It is widely recognized that SAHs cause significant elevations in intracranial pressure. When ICP increases, cerebral perfusion is compromised and cerebral ischemia can occur, potentially triggering insults to the nuclei and affecting the sympathetic nervous system in the brainstem (19, 20). These changes consequently lead to increases in serum catecholamine levels. Previous studies have demonstrated that sympathetic activation and high serum catecholamine concentrations can occur in SAH (18). This observation suggests that elevated serum lactate levels in SAH patients who not have overt hemodynamic disturbances may result from accelerated aerobic glycolysis accompanied by increased production of endogenous catecholamines. In support of this theory, several studies have revealed significant correlations between serum lactate levels and catecholamine concentrations (21, 22). Although it remains unclear which pathophysiologic mechanism contributes the most, both tissue hypoxia-induced anaerobic glycolysis and catecholamine-induced aerobic glycolysis may contribute to the serum lactate level elevation in SAH patients.

The overall outcome of patients with SAH can be determined mainly by the extent of the brain injury that occurs right after initial bleeding, as well as the development of delayed cerebral ischemia and non-neurological organ dysfunction like neurogenic cardiac injury and/or neurogenic pulmonary injury (23–25). The significant burden imposed by the systemic diseases associated with SAH gives rise to diffuse organ dysfunction and harmful anaerobic glycolysis. In addition, higher levels of catecholamines are released in SAH patients with more significant brain injury. These endogenous catecholamines then trigger the release of endothelin and inflammatory cytokines, which can induce cerebral vasospasm, a condition that is related to the development of delayed cerebral ischemia in SAH (26, 27). Furthermore, catecholamine-mediated changes in patients with SAH play a crucial role in the development of non-neurologic medical complications (28). Specifically, one of the well-known neurocardiogenic injuries after SAH is Tako-Tsubo syndrome, which is characterized by left ventricular apical ballooning and transient cardiac dysfunction in relation to increased catecholamine. Although Tako-Tsubo syndrome has been regarded as a transient syndrome, it has been noted that Tako-Tsubo syndrome can significantly contribute to the morbidity and mortality of SAH patients (29).

We have speculated that serum lactate can be a surrogate biomarker for catecholamine release, tissue hypoxia, and hypoperfusion in SAH and that it could be a reliable predictor of SAH severity. This supposition is consistent with previous studies that demonstrated that the serum lactate in SAH may have a predictive role for mortality and disease severity (30–32). On the other hand, another previous study showed that serum lactate did not predict outcomes in these cases (33). Our study supports the use of serum lactate for predicting outcomes in patients with SAH.

This study had several limitations. First, it was a retrospective study, which may have induced selection bias during enrollment. Second, this study had a small sample size and was conducted at a single center, so a well-designed large-cohort prospective study is needed to confirm the observed relationship between serum lactate level and clinical outcomes in SAH. Third, while we were able to show that serum lactate elevation occurred in patients with more severe SAH, we could not fully elucidate the underlying mechanisms that contributed to this increase. Fourth, serum lactate concentration was measured only once, at the time of presentation to the ED. As a result, we could not assess the clinical significance of serial lactate monitoring for prediction of outcomes in SAH. Additional studies are needed to further elucidate how lactate clearance, i.e., the reduction of lactate concentration over time, affects the prognosis of patients with SAH.

## Conclusion

Serum lactate was associated with mortality in patients with spontaneous SAH induced by ruptured aneurysms and may represent a useful biomarker for the early prediction of mortality in SAH patients presenting to the ED.

## Data Availability Statement

The raw data supporting the conclusions of this article will be made available by the authors, without undue reservation.

## Ethics Statement

The studies involving human participants were reviewed and approved by Konkuk University Medical Center Institutional Review Board. Written informed consent for participation was not required for this study in accordance with the national legislation and the institutional requirements.

## Author Contributions

JK and GK established the main conception and designed this study. Each author took part in the collection of data (CO, KL, DH, SP, and KB), in addition to the analysis and interpretation of data (CO, SK, and JK), and/or the writing and correction of the manuscript (CO, JK, and GK). All of the authors of this manuscript have contributed to the work, approved this manuscript, agreed to being listed as an author, and supported its submission to *Frontiers in Neurology*.

## Conflict of Interest

The authors declare that the research was conducted in the absence of any commercial or financial relationships that could be construed as a potential conflict of interest.
