# Supplementary material for: Serum Lactate Could Predict Mortality in Patients With Spontaneous Subarachnoid Hemorrhage in the Emergency Department
Source: Front Neurol. 2020 Sep 4;11:975. doi: 10.3389/fneur.2020.00975 (PMC7499023; doi:10.3389/fneur.2020.00975)
Supplement: Supplementary file 1 [file Table_1.DOCX]

Table 1. Univariate analysis of various factors for predicting mortality in SAH

|  | Univariate analysis | | |
| --- | --- | --- | --- |
|  | OR | 95% CI | *p* value |
| Age (yr) | 1.04 | 1.00 – 1.07 | 0.042 |
| Gender (M) | 1.71 | 0.69 – 4.21 | 0.245 |
| GCS | 0.71 | 0.62 – 0.80 | < 0.001 |
| Hunt-Hess grade | 4.36 | 2.50 – 7.59 | < 0.001 |
| Modified Fisher grade | 6.76 | 2.34 – 19.53 | < 0.001 |
| Aneurysm size (cm) | 0.97 | 0.81 – 1.17 | 0.776 |
| Seizure before presentation | 2.00 | 0.52 – 7.76 | 0.316 |
| SBP (mmHg) | 0.98 | 0.97 – 0.99 | 0.001 |
| HTN | 1.08 | 0.45 – 2.61 | 0.868 |
| DM | 3.93 | 1.08 – 14.2 | 0.037 |
| WBC (cell/ul) | 1.00 | 1.00 – 1.00 | 0.004 |
| CRP (mg/dL) | 1.15 | 0.71 – 1.86 | 0.576 |
| Lactate (mmol/L) | 1.73 | 1.37 – 2.17 | < 0.001 |
| Glucose (mg/dL) | 1.02 | 1.01 – 1.03 | < 0.001 |
| Creatinine (mg/dL) | 1.93 | 1.05 – 3.54 | 0.034 |
